# Performance of Dry-Seeded Rice Genotypes under Varied Soil Moisture Regimes and Foliar-Applied Hormones

**DOI:** 10.3390/plants9040539

**Published:** 2020-04-21

**Authors:** Rajinder Pal, Gulshan Mahajan, Virender Sardana, Bavita Asthir, Bhagirath Singh Chauhan

**Affiliations:** 1 Department of Biochemistry, Punjab Agricultural University, Ludhiana 141004, Punjab, India; rajslohar@pau.edu (R.P.); virsardana@pau.edu (V.S.); basthir@pau.edu (B.A.); 2 The Centre for Crop Science, Queensland Alliance for Agriculture and Food Innovation (QAAFI) and School of Agriculture and Food Sciences (SAFS), The University of Queensland, Gatton, Queensland 4343, Australia; b.chauhan@uq.edu.au

**Keywords:** stay green genotypes, enzymatic activity, harvest index, antioxidant activity, growth regulating substances, leaf area index, yield attributes, oxidative defense mechanism

## Abstract

Plant hormones influence various physiological processes during the growth and development of plants, but their critical roles in influencing yield and antioxidant activities in dry-seeded rice (DSR) have not been adequately explored. This study aims to analyze the performance and antioxidant activity of contrasting genotypes of DSR in response to soil moisture regimes and foliar-applied hormones. The study comprised sixteen treatments that were evaluated under field conditions as per split-plot design in three replications. Treatments comprised combinations of two soil moisture tension regimes (10 kPa and 20 kPa) and two genotypes (PR-111, non-stay-green type and PR-123, stay-green type) applied to the main plots and foliar application of three hormones (gibberellic acid (GA_3_) 40 mg kg^−1^, abscisic acid (ABA) 20 mg kg^−1^, and cytokinin (CK) 40 mg kg^−1^)) and a control (unsprayed) to subplots. The non-stay-green genotype (PR-111) resulted in 34.6% more grain yield (6.48 t ha^−1^) than the stay-green genotype (PR-123) at the lower soil moisture tension regime (SMTR) (10 kPa) due to the increased number of filled grains per panicle and improvement in harvest index (HI). At the higher SMTR (20 kPa), the stay-green genotype (PR-123) produced 26.4% more grain yield (5.21 t ha^−1^) than non-stay green genotype (4.12 t ha^−1^) and showed enhanced superoxide dismutase (SOD) and peroxide dismutase (POD) activity that may have contributed in maintaining sink size through improved chlorophyll content. Grain yield (6.35 t ha^−1^) with foliar-applied GA_3_ (40 mg kg^−1^) at SMTR of 10 kPa was higher by 12.2% and 24.0% than with foliar-applied ABA (20 mg kg^−1^) and unsprayed treatments, respectively. Irrigation application at SMTR of 20 kPa and foliar application of ABA gave 24.1% higher grain yield (5.15 t ha^−1^) than the unsprayed treatment, but it was similar to foliar-applied GA_3_ and CK. This study implied that the stay-green genotype (PR-123) was more suitable under moisture stress conditions (20 kPa) in DSR, as it maintained sink size even under moisture stress conditions by improving dry matter translocation and enhancing SOD and POD activity. The study suggests the need to find out the endogenous level of these plant hormones in rice genotypes under a range of water regimes to develop high yielding and water use efficient genotypes of DSR.

## 1. Introduction

Rice (*Oryza sativa* L.) is the staple diet of more than 90% population of South Asia and also an essential food for a majority of the world’s population [1,2]. In South Asia, transplanting seedlings into puddled soil is the most common method of rice cultivation. This practice of crop planting is highly water, energy, and labor-intensive. In the North-Western Indo-Gangetic Plains of South Asia, rice is grown on over 12.5 million hectares [3], primarily during the summer rainy season and depends heavily on irrigation. High water requirements of puddled transplanted rice (PTR) have resulted in groundwater depletion to alarming levels due to its over-exploitation [4]. Cultivation of PTR has lowered the water table and has increased the economic and energy resource costs required to extract water from deeper aquifer layers [5]. The labor required for cultivation is also becoming scarce and expensive. There is increasing concern that regional food security may be jeopardized in the near future due to the unsustainable water resources, energy, and labor requirements of PTR. There is thus urgent need to devise alternative methods of rice cultivation with reduced dependence upon such intensive resource requirements.

One viable approach is the dry-seeded cultivation of rice where light irrigation of 50–60 mm is applied after seeds are sown into non-saturated fields. With the availability of effective herbicides for weed management, dry-seeded rice (DSR) is increasingly becoming a feasible and cost-effective system of rice cultivation. There is a significant saving in DSR on costs associated with energy and water for puddling, water for maintenance of nursery seedlings, and the establishment of seedlings in main plots and labor for transplanting in comparison to PTR [6,7,8,9]. Direct-seeded rice usually matures earlier than PTR [10]. However, DSR usually suffers from moisture stress at different growth stages, which limits its sink size. The successful cultivation of DSR, therefore, depends on the selection of genotypes and ameliorative measures to maintain its sink size under probable moisture stress at different phases of plant growth [11]. 

Grain yield is positively correlated with the size of the photosynthetic area and its period of availability for the development of yield-forming traits [11]. However, the onset of leaf senescence greatly reduces the assimilate availability from leaves to grains. Leaf senescence is controlled by hormones and is influenced by the internal metabolic activities, as well as environmental conditions prevailing during plant growth and development. Leaf senescence results in the degradation of rubisco and other proteins into amino acids, which are temporarily stored in leaves. Thus, an expanded photosynthesis period assisted by a delayed onset of leaf senescence could extend the availability of assimilates for grain production. Therefore, the stay-green trait for the retention of green leaf area in plants could provide yield improvement in DSR. 

Reduced availability of post-anthesis assimilates limits grain-filling in DSR. Rice roots are confined to the surface of the soil in DSR where they have limited access to nutrients. Plant stress under these conditions results in the increased formation of reactive oxygen species (ROS) owing to an increase in photorespiration and reduction in the availability of assimilates [12]. Vital plant processes such as photosynthesis also induce oxidative stress in DSR [13]. However, enzymatic antioxidants such as superoxide dismutase (SOD), peroxidase (POD), and catalase (CAT) offer an oxidative defense in plants [14]. Osmotic adjustment improves the water uptake and retention and thereby ameliorates the adverse effect of ROS in plants [15].

Plant hormones such as gibberellic acid (GA_3_), abscisic acid (ABA), and cytokinin (CK) are commonly applied for improved stress resilience in plants. Senescence due to chlorophyll and protein breakdown in leaves is delayed in rice through the application of GA_3_ at the grain-filling stage [16]. The exogenous application of ABA promotes stress tolerance in plants [17,18]. Under moisture stress conditions, exogenous application of ABA interacts with membrane phospholipids and enhances membrane stability [19,20] and tolerance in response to oxidative stress in plants through increased activity of antioxidant enzymes [21,22,23] The exogenous application of CK bolsters reduced plant levels caused by its inhibited synthesis under drought conditions [24,25,26,27,28] and supports plant growth due to higher levels of photosynthesis, water use efficiency, and antioxidant metabolism of shoots [26,29,30,31]. Cytokinin has been shown to moderate enzymatic antioxidant activities such as POD, SOD, and CAT in leaves and to activate leaf defenses against abiotic stresses [27,32], as well as protecting leaves from stress-induced oxidation [33]. Delaying senescence by application of plant hormones could be a viable strategy to extend the period of assimilate availability vis-à-vis its partitioning to developing grains and, consequently, improve grain yield of DSR. 

This study was aimed to exploit the genetic potential of stay-green genotype and to understand the role of plant hormones in yield improvement under moisture stress situations.

## 2. Materials and Methods

### 2.1. Experimental Site

This field experiment was conducted at the research farm of Punjab Agricultural University (PAU), Ludhiana, India (30°54′ N, 75°48′ E; 247 m above sea level) for two consecutive years in 2015 and 2016. Prior to the establishment of this experiment, a rice-wheat (*Oryza sativa* L.–*Triticum aestivum* L.) cropping sequence was implemented in this lowland field for five years. A majority of the growing season (mid-June to October) coincides with the region’s monsoonal rains (early July to mid-September). The long term average annual rainfall of Ludhiana is 734 mm, about 85% of which occurs during the monsoon season. The soil of the experimental field was a Fatehpur loamy sand (Typic Ustipsament) with a pH of 7.2, total nitrogen content of 0.042%, organic carbon content of 0.38%, 0.5 N NaHCO_3_ extractable P of 5.2 µg g^−1^, and NH_4_OAc extractable K of 30 µg g^−1^ (average of two years). The groundwater at the experimental site was nonsaline, with a water table depth of more than 25 m. The soil bulk density at the beginning of the experiment was 1.58 Mg m^−3^, with a saturated hydraulic conductivity of 24 mm h^−1^. 

### 2.2. Experimental Design

The experiment comprised 16 treatments (2 × 2 × 4), which were laid out as per split-plot design of experimentation in three replications. The combination of two soil moisture tension regimes (SMTR), viz., 10 and 20 kPa, and two genotypes, viz., PR-111 (non-stay-green type) and PR-123 (stay-green type), comprised the main factor, whereas four treatments of plant hormones (gibberellic acid 40 mg kg^−1^, abscisic acid 20 mg kg^−1^, cytokinin 40 mg kg^−1^, and water spray without plant hormone) comprised the sub-factor. The need for irrigation to the crop as per treatments (10 kPa and 20 kPa) was determined by measuring the soil moisture tension every morning at 09:00 h with a Soilspec Tensiometer (T K System, Healesville, VIC, Australia) installed with its tips at 15-cm soil depth at the tail end of each soil water tension regime treatment plot. All the plots were separated with double bunds to prevent the redistribution of the irrigation flow of water from one plot to another. The size of each subplot was 2 m by 6 m. Up to the first 25 days after sowing (DAS), all plots were irrigated to maintain a soil water tension of about 10 kPa at a soil depth of 15 cm to avoid any water deficit during crop establishment. Thereafter, irrigation of 75-mm depth was applied when soil water tension reached 10 kPa (well-watered) or 20 kPa (water stress). All irrigation treatments were measured using a Parshall flume (Indian Institute of Technology, Roorkee, India) of a 75-mm throat size. The total irrigation water (irrigation + effective rainfall) applied to the crop was 1441 mm at 10 kPa and 1141 mm at 20 kPa during 2015 and 1409 mm at 10 kPa and 1129 mm at 20 kPa during 2016.

### 2.3. Crop Management, Plant Samplings, and Data-Recording

The field for sowing was prepared by cultivating twice with a disc harrow (Unison Exports, Ludhiana, Punjab, India) followed by leveling with a wooden board. Both the genotypes were manually sown at 20-cm row spacing by dibbling seeds at the rate of 25 kg ha^−1^. The field, immediately after sowing, was surface-irrigated with 50 mm of water. Nitrogen was applied at 150 kg ha^−1^ in three equal splits at 2, 5, and 9 weeks after sowing (WAS). In accordance with the PAU recommendation for the rice-wheat cropping system in the state, phosphorus fertilizer was not applied, as the recommended dose (60 kg P_2_O_5_ ha^−1^) had been applied to the preceding wheat crop. Muriate of potash (60% K_2_O) was applied as potassium fertilizer at a rate of 30 kg K_2_O ha^−1^ at the time of sowing. Weeds were managed with the pre-emergence application (2 DAS) of pendimethalin (0.75 kg a.i. ha^−1^) and a post-emergence application (20 DAS) of bispyribac-sodium (25 g a.i. ha^−1^). The weeds that escaped these herbicides were removed manually. As a prophylactic measure, chlorpyriphos (50 g a.i. ha^−1^) was sprayed to control stem borer (*Scirpophaga incertulas*), leaf folder (*Cnaphalocrocis medinalis*), brown plant hopper (*Nilaparva talugens*), and rice hispa (*Dicladispa armigera*). Similalry, propiconazole (62.5 g a.i. ha^−1^) was sprayed to control sheath blight (*Rhizoctoniasolani*), false smut (*Ustilago inoideavirens*), and kernel smut (*Neovossia horrida*). The crop was sprayed twice (20 and 40 DAS) with a 1% ferrous sulfate solution (250 L ha^−1^) to avoid the occurrence of iron deficiency in plants. The crop was harvested at a grain moisture content of 15%−18%. Genotypes PR-111 and PR-123 took 138 and 148 days to attain maturity, respectively.

Plant hormones were applied at anthesis (~89 DAS in PR-111 and ~99 DAS in PR-123) as a foliar spray. The required amount of water for a correct discharge rate and uniform spray to the point of run-off was calculated by calibrating a motorized knapsack spray pump equipped with a hollow cone nozzle. Each plant hormone solution of desired concentration was prepared by dissolving the required quantity of hormone in 20 mL of suitable surfactant such as GA_3_ in ethanol (70%), ABA in methanol (80%), and CK in concentrated hydrochloric acid (37%). These solutions were further diluted to stock concentrations (1000 mL of 1000 ppm) with distilled water and stored at 4 °C. A final spray volume of 5 liters of each plant hormone was prepared by dilution of stock solution of desired concentrations with distilled water. The final concentrations of surfactants used were 0.28, 0.64, and 0.3 ppm of ethanol, methanol, and hydrochloric acid, respectively. The concentration of each plant hormone was standardized in a previously conducted preliminary experiment. Plots as per treatments were sprayed in the late afternoon on a calm day to avoid spray drift. The plots not treated with plant hormone were sprayed with the same amount of water (without the addition of surfactant) used to apply hormone solution in each treatment plot. 

Grain yield was determined from an area of 8.3 m^2^ (1.6 m × 5.2 m) from eight center rows in each plot (excluding the boundary row and 0.4-m area on both sides of each row). The crop was harvested manually, and a small-plot hand-driven thresher was used for threshing. A moisture meter (Riceter L-Handheld Portable Moisture Meter–Rice, Kett, CA, USA) was used to determine the grain moisture content for each plot. Grain yield was expressed in t ha^−1^ at 14% moisture and then calculated at 0% moisture content. The moisture content of straw was determined by oven-drying a 500-g sample at 70 °C for 72 h. The straw yield was also expressed in t ha^−1^ at 0% moisture content. The harvest index (HI) representing the dry grain yield in proportion to total dry biomass (grain + straw) at crop harvest was calculated. A quadrat (0.4 m by 0.5 m) was placed at two random locations in each plot in order to determine panicle density. Plant height and the number of filled grains per panicle at crop maturity were recorded from five randomly selected plants from each plot. A 1000-grain weight was obtained after threshing from a random sample of the bulk produce of each plot. A digital plant canopy imager (CI/110/CI-120, CID, Inc., Camas, WA, USA) was used to obtain a leaf area index (LAI) at anthesis and other growth stages. The LAI was measured at two specific points in every plot with the probe parallel to the crop row. Dry matter translocation (DMT) to grain was calculated as suggested by [34,35].

DMT = DM_aerial_ − (DM_leaf_ + DM_culm_ + DM_chaff_)

DM_aerial_ represents the dry matter (DM) of aerial parts of the plant at anthesis. DM_leaf_, DM_culm_, and DM_chaff_ are the DM of leaves, culms, and chaff, respectively, at maturity.

### 2.4. Plant Samplings for Biochemical Analysis 

Twenty flag leaves were randomly collected from each plot before the foliar application of plant hormones was made and 15 days thereafter. Directly after collection, these leaves were treated with liquid nitrogen for 1 min and subsequently stored at −80 °C in order to estimate biochemical parameters and enzymes at a later date.

#### 2.4.1. Estimation of Activity of Superoxide Dismutase (SOD), Peroxidase (POD), and Malondialdehyde (MDA) Lipid Peroxide Content

The activity of the SOD enzyme was estimated as per the method given by the authors of [36]. Peroxidase (POD) activity was estimated as per the procedure suggested by the authors of [37]. Lipid peroxidation was determined by the procedure proposed by the authors of [38]. 

#### 2.4.2. Proline, Free Amino Acids, and Total Soluble Proteins in Leaves

The accumulation of proline in leaves was determined by the method given by the authors of [39]. Free amino acids were estimated as per the method described by the authors of [40]. The soluble proteins were determined as per the method proposed by the authors of [41]. 

#### 2.4.3. Total Chlorophyll Content

A leaf tissue sample (0.5 g) was incubated in 5 mL of dimethylsulfoxide at 65 °C for 4 h in order to estimate the total chlorophyll content [42]. The absorbance was recorded at 645 and 663 nm. The estimate for the chlorophyll content was obtained using the calculation proposed by the authors of [43]:

Total chlorophyll content (mg g^−1^) = (20.2 × A_645_) + (8.02 × A_663_)

### 2.5. Weather Data

**Figure 1:**
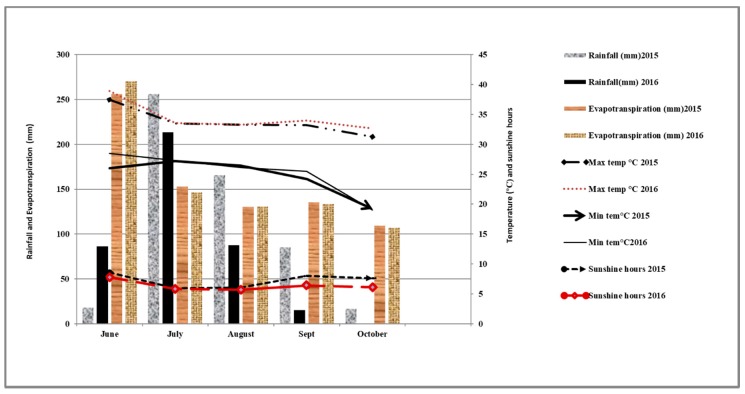
The weather during the crop season (June–October) of 2015 and 2016 measured from the Punjab Agricultural University meteorological station.

The daily minimum and maximum temperatures, hours of sunshine, total rainfall, and evapo-transpiration were recorded at the meteorological station located in PAU at a distance of approximately 200 m from the experimental site. Over the course of the growing season (June–October), total rainfall recorded was 541 mm in 2015 and 409 mm in 2016. The minimum and maximum mean monthly temperatures during the crop growing season in both years did not vary greatly. The average monthly maximum temperature ranged between 31.3 and 37.5 °C in 2015 and between 32.0 and 38.9 °C in 2016. The average monthly minimum temperature during the same period varied from 19.2 and 27.2 °C in 2015 and from 19.0 and 28.5 °C in 2016. The month of June in 2015 experienced a lower mean minimum temperature (26.0 °C) than that of June 2016 (28.5 °C). The mean monthly rainfall in August 2015 was substantially higher (165.6 mm) than in August 2016 (87.6 mm). The number of sunshine hours in September and October 2015 was higher (8.0 and 7.6 h, respectively) than in September and October 2016 (6.4 and 6.1 h, respectively). Pan-evaporation in these respective months of the crop growing season was similar for both years (Figure 1).

### 2.6. Statistical Analysis

Data were subjected to ANOVA using the PROC MIXED procedure of SAS (Statistical Analysis System version 9.2 software, SAS Institute Inc., Cary, NC, USA). The year was considered a random effect, and soil moisture tension regime (SMTR), genotype, and plant hormone were considered as fixed effects in the mixed model analysis. Shapiro–Wilk and Bartlett’s tests were then employed to verify error assumptions such as independence, homogeneity, and normality). In a combined analysis of data, since the interactions of year × SMTR, year × genotype, and year × plant hormones were nonsignificant, the data were pooled over both years. Means for significant effects were separated by LSD, the least significant differences (*p* = 0.05). Due to some laboratory constraints, SOD, POD, and MDA activity was analyzed only in the second year.

## 3. Results and Discussion

### 3.1. Plant Height, Leaf Area Index, and Dry Matter Translocation

The interaction of genotype with a foliar-applied hormone for plant height was significant (Table 1). The plant height of both genotypes (PR-111 and PR-123) was higher with foliar-applied GA_3_ as compared to the control (water spray). Similarly, the plant height of PR-111 with a foliar application of ABA and PR-123 with CK was significantly higher than that attained with control. Plant height with foliar-applied GA_3_ increased by 11% and 15% for PR-111 and PR-123, respectively, whereas it increased by 5% in PR-111 with foliar-applied ABA and 5% in PR-123 with foliar-applied CK, as compared to the water spray treatment. Plants treated with GA_3_ were significantly taller than the untreated (control) plot [44,45]. Gibberellins are responsible for stem elongation, regulate plant growth and development, and enhance the process of cell division in plants [46]. Both genotypes attained similar heights with foliar-applied ABA, whereas, with treatments including water spray, the plants of PR-123 were taller than PR-111. Plant height of PR-123 even with water spray was similar to PR-111 with foliar-applied GA_3_. 

Irrigation application at SMTR of 10 kPa resulted in significantly higher plant height, LAI (at flowering and maturity), DMT, and leaf chlorophyll content at flowering than 20 kPa. Availability of water more frequently throughout the growing season at low SMTR (10 kPa) might have ensured higher plant nutrient uptake via the transpiration stream, which, in turn, might have increased cell elongation and produced higher leaf area at both phonological stages compared to SMTR of 20 kPa. 

Genotype PR-111 attained higher plant height at maturity and LAI at the flowering stage than PR-123. Leaf area index decreased from flowering to maturity. Both genotypes had similar dry matter translocation (Table 2). 

Foliar application of GA_3_ significantly increased the plant height at maturity over the water spray treatment (Table 2). Foliar application of different plant hormones significantly increased the LAI at maturity, DMT, and chlorophyll content (except with GA_3_) as compared to the water spray treatment (Table 2).

Moderate soil drying at the grain-filling stage markedly enhanced grain growth in rice and wheat, which consequently produced higher grain weight, especially in the inferior spikelets [47,48,49], whereas severe moisture stress (50 kPa) imposed during the grain-filling stage significantly reduced grain filling and grain weight in rice because of restricted pre- or post-anthesis dry matter remobilization [50,51]. 

Dry matter translocation with a foliar application of all plant hormones was significantly higher than the water spray treatment (Table 2). Application of ABA resulted in the highest DMT, which was at par with the foliar application of GA_3_ but significantly higher than CK. Dry matter translocation with foliar-applied CK was at par with foliar-applied GA_3_. Kinetin (CK) improves the pre-stored assimilates’ remobilization and regulates the source-sink relationships in plants [52]. Chlorophyll content in the flag leaf decreased from flowering to 15 days after flowering. 

Foliar application of plant hormones retained a higher leaf chlorophyll content at flowering and 15 days later in PR-123 than in PR-111 (Table 2). The net photosynthetic rate in rice genotypes varied during mid- and late-growth stages. The hybrid rice C Liangyou had higher SPAD (soil plant analysis development) values and photosynthetic rates at different growth stages compared with Shanyou 63 [53]. C Liangyou hybrid rice demonstrated an improved yield potential and greater photosynthetic efficiency due to its resistance to leaf senescence and stronger functional leaves [53]. Leaf chlorophyll content at flowering increased with increasing soil moisture tension. Foliar application of ABA and CK demonstrated increased chlorophyll content as compared with water spray at 15 days after flowering. Chlorophyll content significantly enhanced with the application of CK, whereas it significantly reduced with the application of ABA [54]. The spray of GA_3_ reduced the oxidative stress in rice plants grown under stress conditions [55] and augmented the ultra-organizational morphogenesis of plastids to sustain the chlorophyll content and delay senescence [56]. Both ABA and CK are synthesized in roots [57,58] and subsequently transported to above-ground plant parts through the transpiration stream, where these regulate plant growth and development [57,58,59]. The “transpiration pull” required to transport them to above-ground plant parts is governed by SMTR. Plant hormones are also produced in small quantities in the above-ground plant parts [60]. 

The present study indicates that the exogenous application of plant hormones supplemented their endogenous sub-optimal levels and improved the grain yield, as evidenced by the high yield in response to GA_3_ and CK at 10kPa. Similar findings have been reported [61].

Plant growth regulators play a key role in ameliorating the adverse effects of abiotic stresses in crops [62]. Our study revealed that the response varied with the interaction of genotype, moisture regime, and the kind of plant hormone used for foliar spray. Higher moisture stress reduced the endogenous levels of CK in leaves and root exudates in rice and wheat [54,63], which resulted in plant senescence and reduced yield. The lower level of CK in the inferior spikelets (located on proximal secondary branches) reduced the sink size by limiting cell growth in the endosperm and, consequently, reduced the grain weight [54,63]. In the present study, grain yield was reduced when plants were subjected to higher water stress (20 kPa). Increase in the level of CK and GA_3_ with the exogenous application of these hormones at 20 kPa might have promoted the grain filling and extended the period of grain filling in inferior spikelets in rice owing to delayed senescence [51,64] and, thereby, maintained grain yield compared with the water spray treatment. 

### 3.2. Yield, Yield-Contributing Characters, and Harvest Index

**Figure 2:**
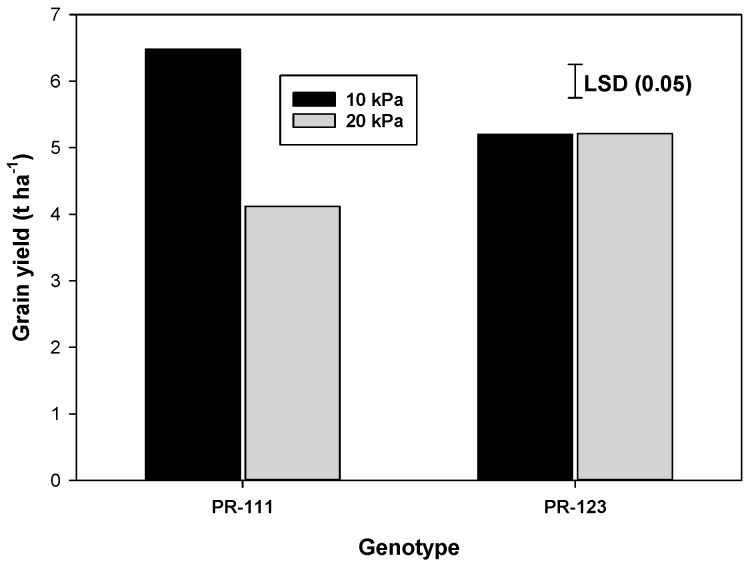

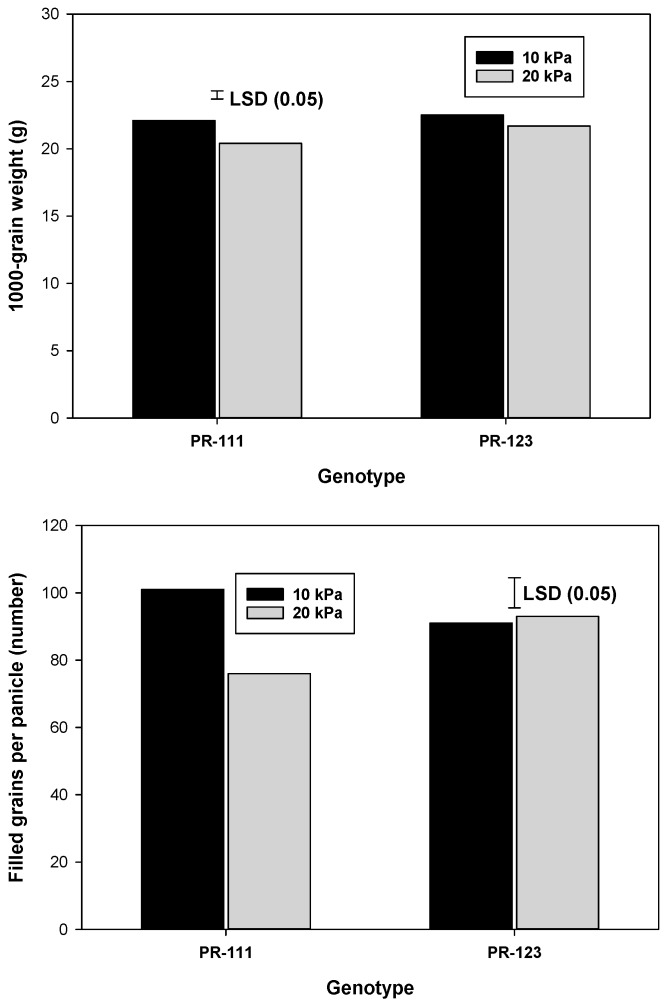
Grain yield, grains panicle^−1^, and 1000-grain weight of rice genotypes (PR-111 and PR-123) under varying irrigation thresholds and foliar applications of plant hormones in dry-seeded rice. PR-111, non-stay-green and PR-123, stay-green.

The interaction between the SMTR and the genotypes for grain yield was significant (Figure 2). At the SMTR of 10 kPa, the grain yield of PR-111 was 24.6% higher than PR-123. However, at the 20 kPa SMTR, PR-111 produced a 20.6% lower grain yield than that of PR-123. Moisture stress imposed at the time of the grain-filling stage enhanced plant senescence and accelerated the grain filling in genotype PR-111 but resulted in slow remobilization of carbon reserved in stem and leaf sheaths in PR-123 (bearing stay-green characteristics). Higher grain yield in PR-111 was attributed to an improved HI as a result of higher LAI at flowering rather than maturity and higher DMT than PR-123 (Figure 2). In comparison, PR-123 attained a lower LAI at flowering and a higher LAI at maturity and translocated lower dry matter to grains and, therefore, had a relatively low HI than PR-111. The grain yield of PR-111 was reduced with increasing soil water tension from 10 kPa to 20kPa; however, for PR-123, it remained similar at both SMTR. PR-111 grown with a soil moisture tension regime of 10 kPa produced a higher number of filled grains per panicle as compared to PR-123, whereas, at SMTR of 20 kPa, PR-123 produced a higher number of filled grains per panicle than PR-111 (Figure 2). With increasing soil water tension from 10 kPa to 20 kPa, filled grains per panicle significantly decreased in PR-111, while, in PR-123, SMTR did not influence grain filling in a panicle. Both the genotypes produced similar 1000-grain weight at SMTR of 10 kPa (Table 3). However, at the SMTR of 20 kPa, PR-123 produced a higher 1000-grain weight than PR-111. The extent of reduction in 1000-grain weight with increasing soil water tension was higher in PR-111 as compared with PR-123 (Figure 2).

The crop attained a higher HI at 10 kPa than at 20 kPa (Table 3). Number of panicles m^−2^ of PR-111 was more than that of PR-123 (Table 3). PR-111 also had a higher HI than PR-123. Grain yield of PR-111 was strongly correlated with LAI at flowering (*r* = 0.91) and maturity (*r* = 0.72) and number of filled grains per panicle at maturity (*r* = 0.96) as compared with PR-123 (*r* = −0.22, 0.21, and 0.86, respectively). It may be ascribed to the remobilization of more quantity of pre-stored DM to the developing grains from the stems and leaf sheaths in PR-111. 

**Figure 3:**
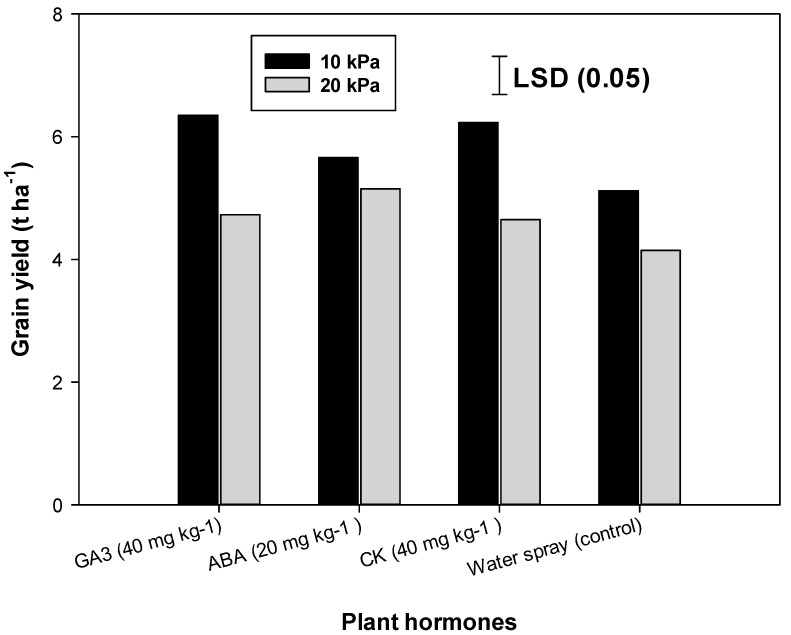
Grain yield (t ha^−1^) of dry-seeded rice (averaged over PR-111 and PR-123) under varying moisture regimes and foliar-applied plant hormones. LSD, least significant differences; GA_3,_ gibberellic acid; CK, cytokinin; and ABA, abscisic acid.

The grain yield was also significantly affected by the interaction of SMTR with a foliar-applied hormone (Figure 3). With increasing soil water tension from 10 kPa to 20 kPa, grain yields were reduced in all hormonal treatments except with foliar-applied ABA. At SMTR of 10 kPa, foliar-applied GA_3_ and CK gave higher grain yields than the water spray treatment, while, at SMTR of 20 kPa, only foliar-applied ABA gave a higher yield than the water spray treatment. Abscisic acid is produced in the plants in response to moisture-deficit situations [54], and it regulates the transportation of stored photosynthates to developing seeds or fruits [65,66,67,68]. Grain yield of crop irrigated at a soil moisture tension of 10 kPa and sprayed with only water was similar to the crop irrigated at a soil moisture tension of 20 kPa and sprayed with ABA (Figure 3). The crop HI with a foliar application of GA_3_ and ABA was higher compared to the foliar-applied CK and water spray treatment.

### 3.3. Antioxidants Activity and Osmoregulation

Interaction effects of SMTR, genotypes, and foliar applications of plant hormones on the proline content, soluble protein, and amino acid in flag leaves at 15 days after a foliar application of plant hormones were significant (Table 4). At SMTR of 10 kPa, flag leaves with foliar-applied GA_3_ had higher proline contents in PR-111 than in the water spray treatment; however, under water stress conditions (20 kPa), a foliar application of ABA and CK registered a higher proline content in the flag leaves of PR-111 than the water spray treatment (Table 4). PR-123 with foliar-applied ABA had a higher proline content than foliar-applied GA_3_ at both SMTR and CK at the SMTR of 20 kPa. PR-123 with a foliar application of CK at the SMTR of 20 kPa also registered significantly lower proline contents in flag leaves than the GA_3_ application. At 10 kPa, proline contents in both genotypes (PR-111 and PR-123) with foliar applications of ABA and CK were similar to that of water-sprayed plants. However, at higher SMTR (20 kPa), foliar application of plant hormones enhanced the proline content in both genotypes except in PR-111 with GA_3_ application in comparison to the water-sprayed treatment; but the percentage increase over the water-sprayed treatment was higher in PR-123 than PR-111. Proline, an amino acid, is reported to act as an osmolyte that scavenges the oxygen-free radicals, thus regulating the osmotic balance of cells and ameliorates the negative effect of moisture stress in plants [69]. Proline gets accumulated in excess when plants are exposed to moisture deficit conditions [70] and, being an electron receptor, protects the photosynthetic machinery under oxidative stress [71]. In our study, the proline content in both genotypes (PR-111 and PR-123) increased in response to the elevated soil moisture tension. PR-111 accumulated more proline at both SMTR compared with PR-123, and the application of ABA and CK augmented it further with an SMTR of 20 kPa. The authors of [72] also reported an increase in proline accumulation when GA_3_ and kinetin were applied under stress conditions, with a markedly higher accumulation of proline in CV-basmati-2000 over KS-133. The authors of [73] reported an enhanced proline concentration with applied GA_3_ and kinetin in many crops. The authors of [74] reported higher proline accumulation in wheat genotypes tolerant to stress in comparison to sensitive genotypes. The authors of [75] also observed variations in proline accumulation among genotypes of rice under moisture stress, with higher proline contents in N-22 at the panicle and anthesis stages relative to ADT 43 and TKM 9. 

Foliar application of water did not influence the soluble protein content of both genotypes at both SMTR (Table 4). However, the soluble protein content in PR-123 with a foliar application of GA_3_ and CK decreased with the increasing soil moisture tension, while, with a foliar application of ABA, the soluble protein contents of genotypes did not vary with the changes of the soil moisture tension. At the SMTR of 10 kPa, the soluble protein content with foliar-applied GA_3_ in both genotypes was higher as compared with the water-sprayed plots. At the SMTR of 10 kPa, a foliar application of ABA, GA_3,_ and CK increased the amino acid content in PR-111 in comparison to the water spray treatment, whereas a similar increase in PR-123 was discerned only with a foliar application of CK over the water spray treatment. At the higher SMTR (20 kPa), a foliar application of CK and GA_3_ increased the amino acid in both genotypes, as compared with the water spray treatment. The amino acid in PR-123 also increased with the ABA application over the water spray treatment (Table 4). Protein accumulated in plants under stress conditions is later utilized by plants for post-stress recovery [76]. This accumulation varied according to genotype, nutrient application, and growth conditions [53,77,78]. Plant metabolism is regulated and promoted by a soluble protein, and its concentration in plants indicates the extent and stage of leaf senescence [79]. 

At an SMTR of 10 kPa, the ABA application improved the SOD activity in both genotypes, as compared to the water-sprayed treatment (Table 5). However, at an SMTR of 20 kPa, a foliar application of all three plant hormones (ABA, GA_3_, and CK) resulted in significantly higher SOD activity in both genotypes in comparison to the water spray treatment, with a greater response discerned with the ABA application. 

In both irrigation regimes, POD activity improved with a foliar application of hormones in comparison to the water-sprayed control (Table 5); a greater response under the SMTR of 10 kPa was, however, observed with GA_3_ for PR-111 and both CK and GA_3_ for PR-123 and with ABA for both genotypes under water stress conditions (20 kPa). The MDA production in flag leaves decreased with foliar-applied hormones in both genotypes and at both SMTR, as compared to the water spray treatment (Table 5). However, such a reduction in MDA production was greater with the ABA application than other hormones (Table 5). The SOD and the POD are important antioxidant enzymes that help to alleviate the adverse effects of reactive oxygen species produced under stress in plants. This study revealed the enhanced activity of SOD and POD enzymes with the application of plant hormones, particularly under soil moisture stress. A greater response was observed with a foliar-applied ABA. Consequently, a higher grain yield was obtained at 20 kPa with a foliar application of ABA as compared to the water spray. The exogenous application of GA_3_, ABA, and CK reduced the production of MDA. The application of plant hormones partially contributed to alleviating the adverse effects of moisture stress by operating the antioxidant system of the plant. The authors of [21] reported higher SOD activity with the application of ABA. Enhanced activity of enzymes such as SOD and POD and reduction in the accumulation of MDA in flag leaves with an exogenous application of 6-BA (cytokinin) at the late-growth stage of rice was reported by the authors of [80]. The authors of [81] observed that an ABA application to a susceptible variety of wheat increased the activity of SOD and POD under a drought. In this present study, the production of SOD and POD in DSR was also influenced by the interaction of genotype and moisture regimes.

### 3.4. Correlation Studies

The grain yield of genotype PR-111 was positively correlated with the number of filled grains per panicle, the 1000-grain weight, and the HI at maturity (Table 6). The LAI at flowering and maturity showed a positive correlation with the plant height at flowering and with grain yield, a number of filled grains per panicle, and the HI. The number of filled grains per panicle was positively correlated with the 1000-grain weight. The grain yield of genotype PR-123 showed a positive correlation with dry matter translocation, the number of filled grains per panicle, and the HI (Table 7). The HI of PR-123 was also positively correlated with the number of filled grains per panicle. This suggested that stay genotypes such as PR-123 resulted in high yields due to better dry matter translocation, as the high dry matter translocation contributed to a high grain number of panicles and ultimately improved the HI.

## 4. Conclusions and Future Implications

This study elucidates the performance of non-stay-green (PR-111) and stay-green (PR-123) rice genotypes under two soil moisture stress regimes (10 and 20 kPa) and the role of plant hormones (gibberellic acid, abscisic acid, and cytokinin) in ameliorating the moisture stress under dry-direct-seeded conditions in North-West India. The stay-green characteristic of the genotype proved more efficient under water-scarce situations in promoting dry matter translocation from vegetative parts to developing grains during the post-anthesis period. This study implied that, under water stress conditions (20 kPa) in DSR, the stay-green genotype (PR-123) performed better by maintaining a sink size through the increased dry matter translocation and enhanced SOD and POD activity. Under the low soil moisture tension regime (10 kPa), the application of GA_3_ and CK improved their endogenous levels and promoted photo assimilation and their translocation towards the developing spikelets. Under a higher soil moisture tension regime, the ABA application promotes the activity of the antioxidant defense mechanism of plants due to the enhanced activity of the SOD, POD, and osmoregulation processes. This study concluded that, under water stress conditions, stay-green genotypes gave a high yield due to the better translocation of assimilates. Further, foliar-applied ABA helped in improving the productivity of rice under water stress conditions. The study suggests the need to develop high-yielding and water use-efficient genotypes of DSR and to find out the endogenous levels of hormones for a range of water regimes.

## Figures and Tables

**Table 1:** Plant height (cm) of dry-seeded rice genotypes at maturity under foliar-applied plant hormones.

Plant Hormones	Plant Height (cm)	
	Genotypes	
	PR-111	PR-123
GA3 (40 mg kg^−1^)	81.3	90.6
ABA (20 mg kg^−1^)	77.0	80.0
CK (40 mg kg^−1^)	76.0	82.7
Water spray (control)	73.3	78.7
LSD_0.05_	3.7	

LSD, least significant differences; GA_3,_ gibberellic acid; CK, cytokinin; and ABA, abscisic acid. PR-111, non-stay-green and PR-123, stay-green.

**Table 2:** Plant height at maturity, dry matter translocation, leaf area index, and chlorophyll content at anthesis and at maturity of genotypes under varying soil moisture tension regimes and foliar-applied plant hormones in dry-direct-seeded rice.

Treatments	Plant Height at Maturity (cm)	Leaf Area Index at Flowering	Leaf Area Index at Maturity	Dry Matter Translocation (Mg per ha)	Total Chlorophyll Leaf (mg per g fw)	
					at Flowering before Spray	at 15 Days after Spray
Soil moisture tension regimes						
10 kPa	82.0	4.30	3.90	2.85	2.87	2.58
20 kPa	78.0	3.70	3.18	2.30	3.02	2.61
LSD_0.05_	3.0	0.11	0.16	0.48	0.09	NS
Genotypes						
PR-111	76.9	4.10	3.48	2.66	2.70	2.35
PR-123	83.0	3.95	3.60	2.47	3.19	2.85
LSD_0.05_	3.0	0.11	NS	NS	0.03	0.06
Plant hormones						
GA_3_ (40 mg kg^−1^)	86.0	4.0	3.63	2.78	2.95	2.58
ABA (20 mg kg^−1^)	78.5	4.0	3.73	3.11	2.93	2.63
CK (40 mg kg^−1^)	78.0	4.0	3.73	2.46	2.95	2.66
Water spray (control)	77.4	4.0	3.10	1.91	2.94	2.52
LSD_0.05_	2.6	NS	0.23	0.50	NS	0.08

Fw, fresh weight; GA_3,_ gibberellic acid; CK, cytokinin; ABA, abscisic acid; Mg, tonne; LSD, least significant differences; and NS, nonsignificant.

**Table 3:** Grain yield, panicles m^−2^, filled grain panicle^−1^, 1000-grain weight, and harvest index of dry direct-seeded rice genotypes under varying irrigation thresholds and foliar-applied plant hormones.

Treatments	Grain Yield (t ha^−1^)	Panicles m^−2^ (Numbers)	Filled Grain per Panicle (Numbers)	1000-Grain Weight (g)	Harvest Index
Soil moisture tension regimes					
10 kPa	5.8	383	96	22.3	0.35
20 kPa	4.7	372	84	21.0	0.30
LSD_0.05_	0.3	NS	6	0.4	0.02
Genotypes					
PR-111	5.3	386	88	21.3	0.35
PR-123	5.2	368	92	22.1	0.31
LSD_0.05_	NS	12	NS	0.4	0.02
Plant hormones					
GA (40 mg kg^−1^)	5.5	388	93	21.5	0.34
ABA (20 mg kg^−1^)	5.4	376	94	22.0	0.35
CK (40 mg kg^−1^)	5.4	380	90	21.8	0.32
Water spray (control)	4.6	366	83	21.3	0.30
LSD_0.05_	0.4	NS	6	0.4	0.02

LSD, least significant differences and NS, nonsignificant.

**Table 4:** Moisture regimes x genotypes x plant hormones interactions for proline, soluble protein, and amino acid contents in flag leaves at 15 days after foliar-applied plant hormones in dry-direct-seeded rice.

Plant Hormones	Soil Moisture Tension Regimes			
	10 kPa		20 kPa	
	Genotypes			
	PR-111	PR-123	PR-111	PR-123
Proline contents ( µg per g of fresh leaves)				
GA_3_ (40 mg kg^−1^)	170.6	129.5	154.5	180.6
ABA (20 mg kg^−1^)	147.8	147.5	214.1	198.8
CK (40 mg kg^−1^)	133.7	144.7	217.4	154.3
Water spray (control)	143.1	138.7	170.6	106.6
LSD_0.05_	16.2			
Soluble protein (%)				
GA_3_ (40 mg kg^−1^)	8.6	9.2	7.8	7.3
ABA (20 mg kg^−1^)	7.7	7.5	7.3	7.1
CK (40 mg kg^−1^)	7.0	8.4	6.9	6.8
Water spray (control)	7.3	5.9	7.0	6.6
LSD_0.05_	0.8			
Amino acids (%)				
GA_3_ (40 mg kg^−1^)	0.59	0.34	0.57	0.61
ABA (20 mg kg^−1^)	0.60	0.46	0.43	0.65
CK (40 mg kg^−1^)	0.59	0.65	0.68	0.62
Water spray (control)	0.40	0.46	0.45	0.52
LSD_0.05_	0.04			

LSD, least significant differences; GA_3,_ gibberellic acid; CK, cytokinin; and ABA, abscisic acid.

**Table 5:** Activity of superoxide dismutase (SOD), peroxidase dismutase (POD), and the formation of malondialdehyde (MDA) under varying moisture regimes, genotypes, and foliar-applied plant hormones in dry-seeded rice.

Plant Hormones	Soil Moisture Tension Regimes			
	10 kPa		20 kPa	
	Genotypes			
	PR-111	PR-123	PR-111	PR-123
Activity of SOD (U per mg of protein)				
GA_3_ (40 mg kg^−1^)	3.71	4.33	5.52	4.75
ABA (20 mg kg^−1^)	4.87	6.16	5.68	5.71
CK (40 mg kg^−1^)	3.75	4.31	3.54	4.21
Water spray (control)	3.52	4.20	2.63	2.40
LSD_0.05_	0.62			
Activity of POD (µ moles per minute per gram of fw)				
GA_3_ (40 mg kg^−1^)	5.62	6.50	6.03	5.99
ABA (20 mg kg^−1^)	5.10	5.12	7.73	6.81
CK (40 mg kg^−1^)	5.03	6.79	7.08	5.35
Water spray (control)	3.92	4.45	4.98	5.01
LSD_0.05_	0.36			
MDA production in flag leaf (mg per g of fw)				
GA_3_ (40 mg kg^−1^)	7.60	9.70	7.20	6.90
ABA (20 mg kg^−1^)	5.70	8.40	5.80	6.00
CK (40 mg kg^−1^)	9.90	12.1	11.80	6.00
Water spray (control)	15.2	14.5	15.4	10.9
LSD_0.05_	1.05			

LSD, least significant differences; GA_3,_ gibberellic acid; CK, cytokinin; and ABA, abscisic acid.

**Table 6:** Correlation among different traits in response to exogenously applied plant hormones and irrigation thresholds for genotype PR-111 (critical value of *r* at 5% level of significance = 0.71).

	GY	P m^−2^	G P^−1^	1000-GW	HI	P. Ht. (F)	LAI (F)	LAI (M)	DMT
GY									
P per m^2^	0.56								
G per P	0.96 *	0.52							
1000-GW	0.98 *	0.51	0.93 *						
HI	0.98 *	0.61	0.98 *	0.95 *					
Pl. Ht (F)	−0.04	−0.20	0.07	0.13	−0.12				
LAI (F)	0.91 *	0.54	0.95 *	0.86 *	0.81 *	0.95 *			
LAI (M)	0.72 *	0.50	0.68	0.77 *	0.69	0.71 *	0.23		
DMT	0.34	0.43	0.30	0.37	0.30	0.39	0.26	0.49	

* Indicates relationship was significant at 5% level of significance. Abbreviations: GY, grain yield; P per m^2^, number of panicles per square meter; G per P, number of grains per panicle; GW, grain weight; HI, harvest index; Pl. Ht. (F), plant height at flowering; LAI (F), leaf area index at flowering; LAI (M), leaf area index at maturity; and DMT, dry matter translocation.

**Table 7:** Correlation among different traits in response to exogenously applied plant hormones and irrigation thresholds for genotype PR-123 (critical value of *r* at 5% level of significance = 0.71).

	GY	P m^−2^	G P^−1^	1000-GW	HI	P. Ht. (F)	LAI (F)	LAI (M)	DMT
GY									
P per m^2^	0.12								
G per P	0.86 *	−0.32							
1000 GW	0.34	−0.58	0.40						
HI	0.85 *	0.03	0.76 *	0.42					
Pl. Ht. (F)	−0.15	−0.07	−0.25	0.28	−0.12				
LAI (F)	−0.22	−0.15	−0.22	0.41	0.27	−0.38			
LAI (M)	0.21	0.00	0.01	0.55	−0.09	0.21	−0.25		
DMT	0.72 *	0.21	0.49	0.47	0.28	0.52	−0.29	0.45	

* Indicates relationship was significant at 5% level of significance. Abbreviations: GY, grain yield; P per m^2^, number of panicles per square meter; G per P, number of grains per panicle; GW, grain weight; HI, harvest index; Pl. Ht. (F), plant height at flowering; LAI (F), leaf area index at flowering; LAI (M), leaf area index at maturity; and DMT, dry matter translocation.
